# The relationship between METS-IR and the risk of diabetes incidence in rural adults in China: A retrospective cohort study based on dynamic population

**DOI:** 10.1371/journal.pone.0341612

**Published:** 2026-01-28

**Authors:** Zihao Li, Xuejiao Chen, Wanli Hu, Gefei Li, Xiaoke Zhang, Datian Gao, Haiyun Gao, Songhe Shi

**Affiliations:** Department of Epidemiology and Health Statistics, College of Public Health, Zhengzhou University, Zhengzhou, Henan, People’s Republic of China; University of Montenegro-Faculty of Medicine, MONTENEGRO

## Abstract

**Objective:**

To evaluate the longitudinal association between the Metabolic Score for Insulin Resistance (METS-IR) and the risk of diabetes mellitus in rural Chinese adults.

**Methods:**

This retrospective cohort study included 53,120 participants aged ≥18 years from 2018 to 2023. Participants were stratified by quartiles of the METS-IR metrics. Cox proportional hazards models assessed the association between METS-IR and incident diabetes. Restricted cubic spline (RCS) models examined nonlinear trends. Subgroup analysis, interaction tests, and multiple sensitivity analyses were performed. Predictive ability was evaluated using time-dependent receiver operating characteristic (ROC) curves.

**Results:**

During 176,413.4 person-years of follow-up (median 3.83 years), 14,397 participants developed diabetes. After multifactorial adjustment, METS-IR was significantly and positively associated with diabetes onset (hazard ratio (HR)=1.094,95% confidence interval (CI): 1.076–1.112, P < 0.001); those in the highest quartile group had a 1.435-fold higher risk compared to the lowest. RCS analysis revealed a nonlinear dose-response relationship. Kaplan-Meier curves confirmed increasing cumulative risk with higher METS-IR. Results remained robust across subgroups and sensitivity analyses. The area under the curve (AUC) for METS-IR predicting diabetes was 0.601 (1 year), 0.586 (3 years), and 0.599 (5 years).

**Conclusion:**

METS-IR is significantly correlated with the onset of diabetes, and the relationship is nonlinear. While it demonstrates limited discriminatory performance as a standalone screening tool, it remains suitable for initial risk stratification in primary health care institutions with limited resources.

## Introduction

Diabetes mellitus (DM) has emerged as a major global public health problem, with its prevalence and incidence continuously rising worldwide and imposing an increasingly heavy public health burden [1] According to the 10^th^ edition of the International Diabetes Federation (IDF) Diabetes Atlas, an estimated 537 million individuals globally will have diabetes in 2021, and this figure is projected to increase to 783 million by 2045 [2]. It is worth noting that adult diabetes patients in the Western Pacific region account for more than one-third (38%) of the total, among which China makes up one-quarter of the global total [3]. The number of patients in China is estimated to have exceeded 140 million in 2021, and this number is predicted to climb to 174 million by 2045 [2]. It is well known that if diabetes is not effectively controlled and treated, it is highly susceptible to multi-system damage, inducing complications such as cardiovascular disease, renal function impairment, and retinopathy [4–7]. Studies have predicted that the economic burden related to diabetes in China will increase from 1.58% of GDP to 1.69% between 2020 and 2030 [8]. With the increasing prevalence of diabetes and its economic burden, early detection and intervention of diabetes has become an important topic that requires urgent attention.

Insulin resistance (IR) is a common feature in the early stages of both diabetes and hypertension, and it is also an important precursor to their development [9]. Existing studies have shown that IR is not only an important driver of diabetes development, but also significantly increases the risk of dyslipidemia, hypertension, and coronary artery disease [10,11]. Therefore, early intervention at the pre-diabetic and/or insulin resistance stages can help reduce the burden of disease and healthcare costs for individuals and society [12–14].

The gold standard for assessing insulin resistance is usually the hyperinsulinemic-euglycemic clamp (HEC) [15]. However, despite its high accuracy, it has not been widely promoted and applied because of its complex operation, time-consuming nature, expense, and invasiveness [16]. Meanwhile, fasting insulin-based indices, such as the homeostasis model assessment of IR (HOMA-IR and quantitative insulin sensitivity check index (QUICKI), although widely used, still suffer from high variability and limited practical application due to their dependence on insulin measurement [17]. These challenges highlight a key limitation: the dependence on complex procedures or non-standardized insulin assays restricts large-scale IR screening. Therefore, to overcome these limitations, researchers have developed a series of non-insulin-based IR assessment indices, such as triglyceride to high-density lipoprotein cholesterol ratio (TG/HDL-C), triglyceride glucose index (TyG index), and triglyceride glucose-body mass index (TyG-BMI) [18–20]. These indices, which use routinely collected metabolic parameters, have gained broad application in epidemiological research and clinical screening.

Recently, building upon this foundation of non-insulin-based indices, a new metabolic score for insulin resistance (METS-IR) has emerged, which comprehensively considers parameters such as fasting plasma glucose (FPG), lipid profile, and obesity index. As a non-insulin-based indicator with improved stability and convenience, METS-IR has become an important tool for evaluating insulin sensitivity [16]. Studies have shown that in predicting insulin resistance, METS-IR has higher accuracy than TyG and TG/HDL-C, and performs well in predicting visceral fat accumulation and type 2 diabetes [16]. However, the relationship between METS-IR and the development of diabetes, especially in large sample populations, is still relatively limited and needs to be further explored. Therefore, this study aims to explore the relationship between baseline METS-IR levels and the risk of developing diabetes in a large cohort of adults from rural China. The findings are expected to provide scientific evidence for the early identification and prevention of diabetes.

## Methods

### Study design and participants

This is a retrospective cohort study based on a dynamic population, with data from an annual health check-up program implemented in Xinzheng City, Henan Province, since 2010. The research data is derived from the electronic health records of residents in the information system of Xinzheng Hospital from January 1, 2018 to December 31, 2023. This de-identified data was accessed for research purposes beginning on March 1, 2025. All residents aged ≥18 years who participated in the annual health check-up were considered eligible for cohort entry. New participants could enroll in any year during the study period. Participants could exit the cohort for reasons such as out-migration from the study area or death. To preserve the accuracy of the cohort data, only one health check-up record was kept for each participant per year. We took the time of entry into the cohort as the starting point for follow-up. Individuals were only considered at risk from this entry time onward, ensuring proper handling of left-truncated data. For participants who had no diabetes events, were lost to follow-up, or died at the end of the study period, their data were regarded as right censoring. The details of this dynamic cohort have been described in detail in previous studies [21,22]. All participants received standardized questionnaires, anthropometric measurements, and laboratory tests at baseline and follow-up. During the study period, a total of 491,481 participants aged ≥18 years were enrolled. According to the research design, we excluded 438,361 individuals who met any of the following criteria (S1 Fig): those with only one physical examination record (n = 375,028); those with diagnosed diabetes mellitus at baseline, fasting plasma glucose (FPG) ≥ 126 mg/dL, or glycated hemoglobin A_1c_ (HbA_1c_) ≥ 6.5% (n = 37,371); those with waist circumference (WC), body mass index (BMI), lipid total cholesterol (TC), lipid triglycerides (TG), high-density lipoprotein cholesterol (HDL-C), low-density lipid cholesterol (LDL-C), FPG, systolic blood pressure (SBP), diastolic blood pressure (DBP), smoking, drinking, and physical activity with missing information (n = 25,812); and those with abnormal waist circumference data at baseline (WC < 63 cm for men and WC < 58 cm for women, n = 150). Ultimately, a total of 53,120 participants aged ≥18 years were included in this study, which included information on the timing of diabetes events during their follow-up period.

### Data collection

Data were collected through standardized questionnaires, physical examinations, and laboratory tests. The standardized questionnaire was based on the National Norms for Basic Public Health Services (Third Edition), and its content includes sociodemographic characteristics (age and gender), medical history (including type 2 diabetes, hypertension, coronary heart disease, stroke, chronic obstructive pulmonary disease, cancers, and severe psychiatric illnesses), smoking, drinking, and physical activity, and was filled out by a trained researcher. Based on the self-reported marital status, smoking, and drinking habits of the subjects, participants were classified as married or other; never smokers, ex-smokers, or current smokers; and never, occasional, regular, or daily drinkers. Physical activity frequency was described as daily, more than once a week, occasional, and never.

While standing, participants stood barefoot and measured height and weight to the nearest 0.1 cm and 0.1 kg, with results averaged over two repetitions. The waist circumference was measured at the lowest costal margin and the midpoint of the iliac crest to the nearest 0.1 cm, following the standard procedure. BMI is calculated by dividing weight (in kilograms) by the square of height (in meters), while the waist-to-height ratio is calculated by dividing waist circumference (in centimeters) by height (in centimeters). Blood pressure was measured at least twice using an automatic sphygmomanometer (OMRON HEM-7125, Kyoto, Japan), and the mean of the two measurements was used for analysis [23]. Blood samples were collected for laboratory testing after subjects had fasted for 8 hours or longer, and fasting blood glucose levels were assessed using an automated biochemical analyzer (DIRUI CS380, Changchun, China) [24].

### Definitions

In this study, the diagnostic criteria for diabetes are one of the following: (1) self-reported doctor diagnosed it as diabetes; (2) FPG ≥ 126 mg/dL; (3) HbA_1c_ ≥ 6.5%. Patients with fasting plasma glucose (FPG) between 6.1 and 6.9 mmol/L are defined as prediabetic according to the diagnostic criteria of the World Health Organization (WHO) [25]. The METS-IR was calculated as follows:


METS−IR=Ln[(2×FPG(mg/dL))+TG(mg/dL)]×BMI(kg/m²)/(Ln[HDL−C(mg/dL)])

### Statistical analysis

First, all participants were divided into four quartile groups by baseline METS-IR level. Normality of continuous variables was assessed by the Kolmogorov-Smirnov test. Variables that conformed to a normal distribution were expressed as mean ± standard deviation (Mean ± SD); non-normally distributed variables were expressed as median (interquartile range). Categorical variables are presented as frequencies (percentages, %). For the inter-group comparisons of continuous variables, Student’s t-test was used for those with normal distribution; the Kruskal-Wallis test was used for those with non-normal distribution. For comparisons of categorical variables, the chi-square test was used. The association between METS-IR and the risk of developing diabetes was assessed by univariate and multivariate Cox proportional hazards regression models.

Kaplan-Meier survival analysis was used to plot survival curves for cumulative diabetes incidence, and the differences among different METS-IR quartile groups were evaluated by log-rank test.

The study population was divided into first, second, third, and fourth quartile groups according to the level of METS-IR, and also analyzed with METS-IR per SD increment as a continuous variable. The following four Cox regression models were constructed to assess the association between different METS-IR levels and the risk of diabetes mellitus, and the HR and its 95% CI were calculated, including the original model: unadjusted for any variables; Model I: adjusted for age, gender, and marital status; Model II: based on Model I, further adjusted for SBP, DBP, and LDL-C; Model III: based on Model II, further incorporating BMI, WC, frequency of exercise, alcohol consumption status, and smoking status. All Cox proportional hazards regression models verified the proportional hazards hypothesis through the Schoenfeld residual test. The test results show that all proportional risk assumptions hold true. Furthermore, based on Model Ⅲ, a restricted cubic spline (RCS) with four knots positioned at the 25th, 50th, 75th, and 95th percentiles was used to fit the dose-response relationship between METS-IR levels and the risk of diabetes development to further assess the nonlinear trend. The reference value of the hazard ratio (HR) is set to 1, and the corresponding METS-IR reference point is the median of its distribution. First, examine the overall correlation. If the overall correlation test is significant, further verify the results of the nonlinear and linear tests. When the P-value of the nonlinear correlation is less than 0.05, it indicates that the result is significant and there is a nonlinear correlation.

In addition, stratified analyses and interaction tests were performed based on the following variables to assess the potential heterogeneity and interaction effects between METS-IR levels and diabetes onset in different subgroups: age (18–66 years versus ≥66 years), gender (male versus female), marital status (married versus other), frequency of exercise (low versus high), and frequency of alcohol consumption (low versus high).

Further, the robustness of the association between METS-IR and the risk of developing diabetes mellitus was verified by multiple sensitivity analyses, specifically including: Sensitivity analysis-1: subjects with a BMI < 18.5 kg/m² were excluded; Sensitivity analysis-2: on the basis of sensitivity analysis-1, subjects with a follow-up period of less than 24 months were further excluded to reduce the possibility of reverse causation, as early incident diabetes may reflect pre-existing but undiagnosed disease rather than exposure-related risk; Sensitivity analysis-3: on the basis of sensitivity analysis-2, further excluded subjects with hypertension, chronic obstructive pulmonary disease, coronary heart disease, stroke, cancer, and severe mental illness at baseline. Finally, time-dependent ROC curve analysis using the timeROC package in R (package: timeROC) was used, and the AUC was calculated to assess the predictive ability of the METS-IR for the risk of developing diabetes mellitus at 1 year, 3 years, and 5 years. The analysis incorporated inverse probability of censoring weights to account for right-censoring.

All statistical analyses of data were done using R-project (version 4.3.1). All statistical tests were two-sided, and P values <0.001 were considered statistically significant, which represents a conservative threshold adopted due to the large sample size of this study.

### Ethics approval

The study was conducted according to the guidelines of the Declaration of Helsinki and was approved by the Ethics Committee of Zhengzhou University (Reference Number: ZZUIRB2019−019), written informed consent was obtained from all participants.

## Results

### Baseline characteristics

A total of 53,120 participants (25,908 males and 27,212 females) were enrolled in the study, 48.8% males and 51.2% females, with a median age of 66.0 years (63.0,72.0). During 176,413.4 person-years of follow-up (median follow-up time of 3.83 years), 14,397 participants (accounting for 27.1%) were diagnosed with new-onset diabetes. They were divided into four groups (Q1-Q4) based on METS-IR quartiles, each containing 13,280 individuals. Table 1 demonstrates the baseline characteristics of participants grouped by METS-IR. With the increase of METS-IR level, indicators such as body weight, BMI, WC, TC, TG, FPG, LDL-C, SBP, DBP, TyG, TYG-BMI, TYG-WC, LAP, VAI, and TG/HDL-C all showed an upward trend (S1 Table), while the level of HDL-C decreased (all P values <0.001). Compared with the METS-IR low quartile group, the METS-IR high quartile group had a significantly higher proportion of females and a higher proportion of married people compared to the other groups. Meanwhile, participants in this group had more current alcohol drinkers, fewer smokers, and a higher prevalence of hypertension but a lower prevalence of chronic obstructive pulmonary disease (COPD).

**Table 1:** The baseline characteristics of participants.

	METS-IR quartiles				
Variables	Q1(n = 13280)	Q2(n = 13280)	Q3(n = 13280)	Q4(n = 13280)	*P* value
Age (years)	68.0 (64.0, 74.0)	66.0 (63.0, 72.0)	66.0 (62.0, 71.0)	65.0 (61.0, 70.0)	<0.001
Height (cm)	158.5 (152.4, 165.0)	159.0 (153.0, 165.0)	159.0 (153.0, 165.0)	158.5 (152.4, 165.0)	<0.001
Weight (kg)	52.8 (48.0, 58.0)	60.0 (55.0, 65.0)	64.8 (59.7, 70.0)	72.0 (65.7, 78.5)	<0.001
BMI (kg/m²)	21.1 (19.8, 22.2)	23.6 (22.7, 24.7)	25.7 (24.5, 26.8)	28.5 (26.9, 30.3)	<0.001
WC (cm)	80.0 (75.0, 84.0)	84.0 (80.0, 88.0)	88.0 (84.0, 92.0)	94.0 (89.0, 100.0)	<0.001
TC (mg/dl)	179.8 (155.8, 204.9)	180.6 (155.8, 204.9)	182.5 (158.6, 208.0)	184.5 (159.7, 210.0)	<0.001
TG (mg/dl)	86.8 (65.5, 114.3)	103.6 (77.9, 134.6)	119.6 (90.3, 160.3)	153.2 (112.5, 211.7)	<0.001
FPG (mg/dl)	86.4 (77.9, 94.3)	88.6 (80.8, 96.7)	90.0 (82.3, 97.7)	91.1 (83.0, 98.8)	<0.001
HDL-C (mg/dl)	60.7 (51.8, 71.5)	53.0 (46.4, 61.5)	49.1 (42.5, 56.8)	42.5 (35.6, 50.3)	<0.001
LDL-C (mg/dl)	97.1 (79.7, 115.2)	99.8 (83.1, 117.6)	100.9 (83.9, 120.3)	101.7 (83.5, 121.4)	<0.001
SBP (mmHg)	131.0 (120.0, 146.0)	134.0 (122.0, 149.0)	137.0 (125.0, 151.0)	140.0 (128.0, 154.0)	<0.001
DBP (mmHg)	78.0 (70.0, 85.0)	80.0 (72.0, 87.0)	80.0 (74.0, 90.0)	83.0 (76.0, 90.0)	<0.001
METS-IR	28.9 (27.0, 30.3)	33.6 (32.6, 34.6)	37.7 (36.6, 38.8)	43.8 (41.7, 47.2)	<0.001
Gender (n, %)					
Male	6702 (50.5)	6666 (50.2)	6401 (48.2)	6139 (46.2)	<0.001
Female	6578 (49.5)	6614 (49.8)	6879 (51.8)	7141 (53.8)	
Marital (n, %)					
Others	2830 (21.3)	2245 (16.9)	2013 (15.2)	1838 (13.8)	<0.001
Married	10450 (78.7)	11035 (83.1)	11267 (84.8)	11442 (86.2)	
Exercise (n, %)					
Daily	2813 (21.2)	2978 (22.4)	3357 (25.3)	3230 (24.3)	<0.001
Frequent	726 (5.5)	868 (6.5)	878 (6.6)	701 (5.3)	
Occasional	393 (3.0)	409 (3.1)	454 (3.4)	415 (3.1)	
Never	9348 (70.4)	9025 (68.0)	8591 (64.7)	8934 (67.3)	
Smoking (n, %)					
Never	10964 (82.6)	11193 (84.3)	11250 (84.7)	11335 (85.4)	<0.001
Former	194 (1.5)	220 (1.7)	216 (1.6)	219 (1.6)	
Current	2122 (16.0)	1867 (14.1)	1814 (13.7)	1726 (13.0)	
Drinking (n, %)					
Never	12414 (93.5)	12429 (93.6)	12327 (92.8)	12237 (92.1)	<0.001
Occasional	519 (3.9)	526 (4.0)	593 (4.5)	616 (4.6)	
Frequent	136 (1.0)	145 (1.1)	161 (1.2)	201 (1.5)	
Daily	211 (1.6)	180 (1.4)	199 (1.5)	226 (1.7)	
Hypertension (n, %)	9393 (70.7)	10266 (77.3)	10963 (82.6)	11565 (87.1)	<0.001
COPD (n, %)	371 (2.8)	206 (1.6)	188 (1.4)	170 (1.3)	<0.001
SMI (n, %)	212 (1.6)	212 (1.6)	188 (1.4)	238 (1.8)	0.112
Cancer (n, %)	20 (0.2)	20 (0.2)	14 (0.1)	7 (0.1)	0.057
CHD (n, %)	2007 (15.1)	1857 (14.0)	1965 (14.8)	2139 (16.1)	<0.001
Stroke (n, %)	243 (1.8)	267 (2.0)	320 (2.4)	297 (2.2)	0.006

Abbreviations: BMI, body mass index; WC, waist circumference; TC, total cholesterol; TG, triglyceride; FPG, fasting plasma glucose; HDL-C, high-density lipoprotein cholesterol; LDL-C, low-density lipid cholesterol; SBP, systolic blood pressure; DBP, diastolic blood pressure; METS-IR, metabolic score for insulin resistance; COPD, chronic obstructive pulmonary disease; SMI, severe mental illness; CHD, coronary heart disease.

### Kaplan-Meier analysis of diabetes

**Fig 1:**
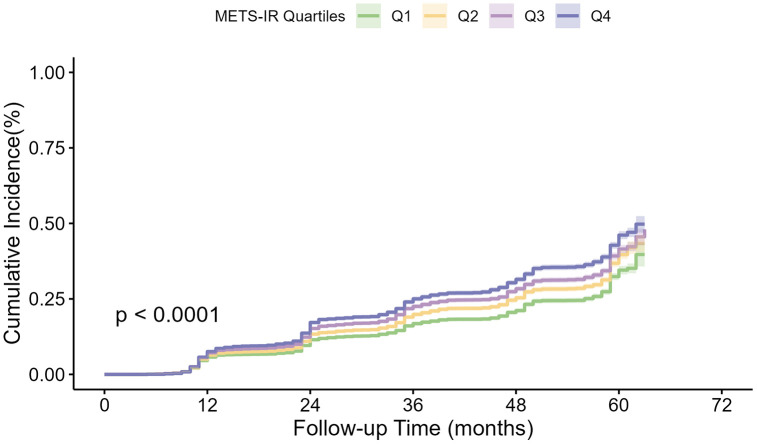
Kaplan–Meier analysis of cumulative incidence of diabetes based on METS-IR quartiles (*P* < 0.0001). Abbreviation: METS-IR, the metabolic score for insulin resistance.

Fig 1 illustrates the Kaplan-Meier curves for the cumulative incidence of diabetes based on baseline METS-IR quartiles. The results showed that quartile group 4 had the highest cumulative incidence of diabetes, while quartile group 1 had the lowest (log-rank test P value < 0.001). This indicates that the higher the level of METS-IR, the greater the risk of developing diabetes.

### Relationship between METS-IR and risk of developing diabetes

In Table 2, the relationship between METS-IR and the risk of developing diabetes is demonstrated. In the unadjusted model (i.e., the original model), for each increase of one standard deviation unit in the baseline METS-IR index, the risk of developing diabetes increased by 1.118-fold (HR = 1.118,95%CI: 1.106–1.131, P < 0.001). In model I, after adjusting for age, gender, and marital status, the HR was 1.132 (95% CI: 1.12–1.143, P < 0.001). In model II, after further adjustment for SBP, DBP, and LDL-C, the HR was 1.128 (95%CI: 1.116–1.14, P < 0.001). Finally, in the fully adjusted model III, the results decreased slightly, with no significant change in the magnitude or direction of the results, and the hazard ratio for diabetes onset was 1.094 (HR = 1.094,95%CI: 1.076–1.112, P < 0.001). Furthermore, when METS-IR was used as a categorical variable and divided into quartiles, the risk of developing diabetes increased with increasing levels of METS-IR, using quartile group 1 as a reference (P < 0.001). In model III, the risk of onset was elevated by 43.5% in quartile group 4 compared to quartile group 1 (HR = 1.435,95%CI: 1.336–1.54, P < 0.001). This series of results all show that there is a significant positive association between the METS-IR index and the occurrence of diabetes.

**Table 2:** Association between METS-IR and the incidence of diabetes in different models.

	No. of cases	Incidence rate^a^	HR (95%CI)			
			Crude Model	Model Ⅰ	Model Ⅱ	Model Ⅲ
METS-IR			1.118 (1.106-1.131)	1.132 (1.12-1.143)	1.128 (1.116-1.14)	1.094 (1.076-1.112)
METS-IR (quartiles)						
Q1	2909	65.29	Reference	Reference	Reference	Reference
Q2	3393	77.2	1.188 (1.131-1.249)	1.228 (1.169-1.291)	1.216 (1.157-1.278)	1.197 (1.136-1.262)
Q3	3779	86.14	1.325 (1.263-1.391)	1.394 (1.328-1.464)	1.37 (1.304-1.438)	1.298 (1.221-1.38)
Q4	4316	98.01	1.508 (1.438-1.58)	1.637 (1.561-1.717)	1.593 (1.518-1.672)	1.435 (1.336-1.54)

^a^per 1000 person-years.

Crude Model: unadjusted for any variables.

Model I: adjusted for age, gender, and marital status.

Model II: adjusted for age, gender, marital status, SBP, DBP, and LDL-C.

Model III: adjusted for age, gender, marital status, SBP, DBP, LDL-C, BMI, WC, frequency of exercise, alcohol consumption status, and smoking status.

Abbreviations: BMI, body mass index; WC, waist circumference; SBP, systolic blood pressure; DBP, diastolic blood pressure; LDL-C, low-density lipoprotein cholesterol.

### Nonlinear analysis

**Fig 2:**
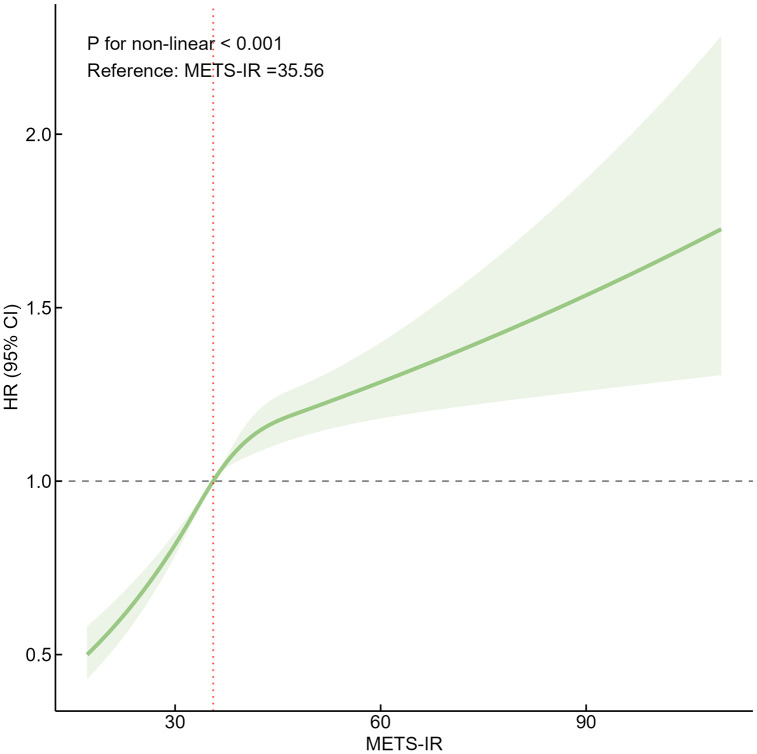
Restricted cubic spline estimates of the relationship between METS-IR and incidence of diabetes. The model was adjusted for age, gender, marital status, SBP, DBP, LDL-C, BMI, WC, frequency of exercise, alcohol consumption status, and smoking status. The model was fitted with knots at the 25th, 50th, 75th, and 95th percentiles of the METS-IR distribution. The green shaded area represents the 95% confidence interval. Abbreviations: METS-IR, the metabolic score for insulin resistance; HR, hazard ratio, CI, confidence interval; BMI, body mass index; WC, waist circumference; SBP, systolic blood pressure; DBP, diastolic blood pressure; LDL-C, low-density lipoprotein cholesterol.

To further explore the dose-response relationship between METS-IR and the risk of developing diabetes, we fitted curves using the RCS model and adjusted for age, gender, marital status, SBP, DBP, LDL-C, BMI, WC, exercise frequency, alcohol consumption status, and smoking status. As shown in Fig 2, there was a significant nonlinear relationship between the two (*P*_nonlinear_<0.001)

### Stratified analysis

In addition, further subgroup analyses were performed to assess the consistency of the association between METS-IR and diabetes under different population characteristics. Table 3 shows that the subgroups were stratified by age (18−66 years versus ≥66 years), gender, marital status (married versus other), exercise frequency (low versus high), and drinking status (low versus high), respectively. And adjusting for confounders such as age, gender, marital status, BMI, WC, exercise frequency, drinking status, and smoking status, HR and 95% CI were calculated for each one SD increase in METS-IR in each subgroup. The results showed that for each SD increase in METS-IR level, the risk of diabetes in women was increased by 12.4% (HR: 1.124, 95%CI: 1.089–1.161), whereas the risk of developing diabetes in men would only increase by 8.4% (HR: 1.084, 95%CI: 1.062–1.106), and the risk of developing diabetes in women was 4% higher than that in men. In subgroup analyses of different ages, for every one standard deviation increase in METS-IR, the increase in the risk of diabetes was comparable between the population < 66 years old (HR: 1.087, 95% CI: 1.054–1.121) and the population ≥ 66 years old (HR: 1.087, 95% CI: 1.065–1.110). From the perspective of different marital statuses, for each increase of one SD in the METS-IR level, the risk of onset in the population other than married (HR: 1.168, 95%CI: 1.109–1.229) was 8.1% higher than that in the married population (HR: 1.087, 95%CI: 1.067–1.106). And from the perspective of different exercise frequencies, for each increase of one SD in the METS-IR level, the population with a low exercise frequency (HR: 1.105, 95%CI: 1.081–1.129) had a 2.6% higher risk of developing the disease than the population with a high exercise frequency (HR: 1.079, 95%CI: 1.052–1.106). Finally, from the perspective of different drinking statuses, at each SD increase in the level of METS-IR, the risk of morbidity was 16.6% higher in those with a high frequency of alcohol consumption (HR: 1.258, 95%CI: 1.089–1.452) than in those with a low frequency of alcohol consumption (HR: 1.092, 95%CI: 1.074–1.111).

**Table 3:** Association between METS-IR and incidence of diabetes in various subgroups.

Subgroup	HR (95%CI)	P value	HR (95%CI)	P value	P for interaction
Gender	female		male		0.38
Q1	Reference		Reference		
Q2	1.226 (1.136–1.323)	<0.001	1.171 (1.088–1.26)	<0.001	
Q3	1.358 (1.244–1.481)	<0.001	1.243 (1.141–1.355)	<0.001	
Q4	1.467 (1.327–1.623)	<0.001	1.405 (1.271–1.554)	<0.001	
Continuous (per SD)	1.124 (1.089–1.161)		1.084 (1.062–1.106)	<0.001	
Age (years)	<66		≥66		0.782
Q1	Reference		Reference		
Q2	1.208 (1.103–1.324)	<0.001	1.165 (1.092–1.243)	<0.001	
Q3	1.329 (1.201–1.471)	<0.001	1.234 (1.142–1.333)	<0.001	
Q4	1.414 (1.26–1.587)	<0.001	1.37 (1.251–1.499)	<0.001	
Continuous (per SD)	1.087 (1.054–1.121)		1.087 (1.065–1.11)	<0.001	
Marital	others		married		0.664
Q1	Reference		Reference		
Q2	1.18 (1.05–1.326)	0.005	1.203 (1.134–1.277)	<0.001	
Q3	1.255 (1.09–1.445)	0.002	1.309 (1.223–1.401)	<0.001	
Q4	1.483 (1.251–1.758)	<0.001	1.431 (1.323–1.548)	<0.001	
Continuous (per SD)	1.168 (1.109–1.229)		1.087 (1.067–1.106)	<0.001	
Exercise	low		high		0.186
Q1	Reference		Reference		
Q2	1.19 (1.118–1.267)	<0.001	1.21 (1.097–1.334)	<0.001	
Q3	1.301 (1.209–1.399)	<0.001	1.287 (1.15–1.44)	<0.001	
Q4	1.413 (1.298–1.539)	<0.001	1.484 (1.304–1.689)	<0.001	
Continuous (per SD)	1.105 (1.081–1.129)		1.079 (1.052–1.106)	<0.001	
Drinking	low		high		0.417
Q1	Reference		Reference		
Q2	1.2 (1.137–1.266)	<0.001	1.12 (0.813–1.543)	0.488	
Q3	1.305 (1.227–1.389)	<0.001	1.177 (0.819–1.691)	0.379	
Q4	1.436 (1.336–1.544)	<0.001	1.463 (0.969–2.208)	0.07	
Continuous (per SD)	1.092 (1.074–1.111)		1.258 (1.089–1.452)	0.002	

Adjusted for age, gender, marital status, SBP, DBP, LDL-C, BMI, WC, frequency of exercise, alcohol consumption status, and smoking status.

Abbreviations: BMI, body mass index; WC, waist circumference; SBP, systolic blood pressure; DBP, diastolic blood pressure; LDL-C, low-density lipoprotein cholesterol; METS-IR, the metabolic score for insulin resistance; SD, standard deviation; HR, hazard ratio; CI, confidence interval.

**Fig 3:**
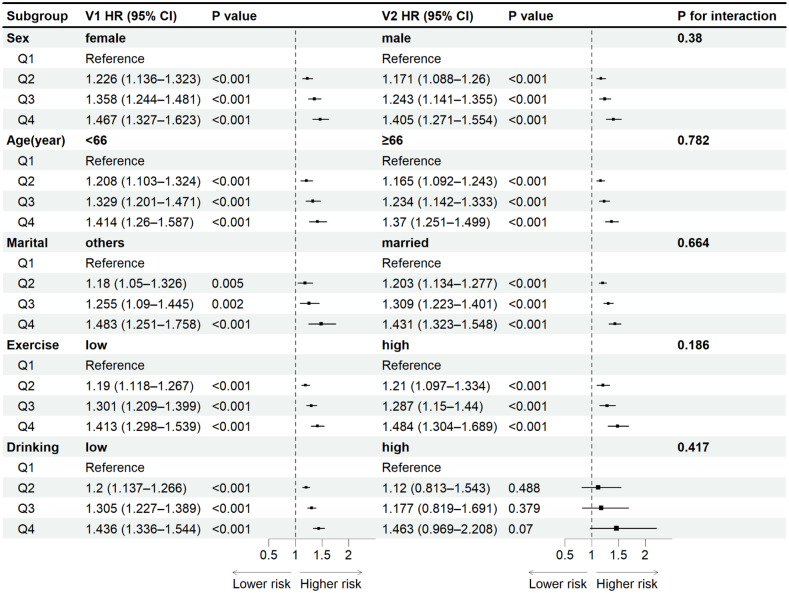
Forest plots of the association between METS-IR and incidence of diabetes in various subgroups. Adjusted for age, gender, marital status, SBP, DBP, LDL-C, BMI, WC, frequency of exercise, alcohol consumption status, and smoking status. Abbreviations: BMI, body mass index; WC, waist circumference; SBP, systolic blood pressure; DBP, diastolic blood pressure; LDL-C, low-density lipoprotein cholesterol; METS-IR, the metabolic score for insulin resistance; HR, hazard ratio; CI, confidence interval.

Overall, the results of all subgroup analyses maintained a consistent association with the results of the main analyses (Table 3); that is, elevated METS-IR was significantly associated with an increased risk of developing diabetes. (P < 0.001). When METS-IR was similarly increased by one standard deviation unit, females, unmarried individuals, those with a lower frequency of exercise, and those who consume alcohol frequently have a higher risk of diabetes onset. No significant interactions (Fig 3) were found between age, sex, marital status, frequency of exercise, and alcohol consumption status with METS-IR and diabetes onset (all *P*_interaction_ >0.05). This suggests that the relationship between METS-IR and diabetes onset is not influenced by these factors.

### Sensitivity analyses

To further validate the robustness of the results, based on the three models mentioned above, we conducted three sensitivity analyses. The results show that the direction and magnitude of the association between METS-IR and the risk of diabetes remained consistent regardless of the exclusion strategy, suggesting that the results of our study have good stability and reliability. The detailed hazard ratios and 95% confidence intervals for all sensitivity models are presented in Supplemental S2 Table.

### Predictive ability of METS-IR for diabetes

**Fig 4:**
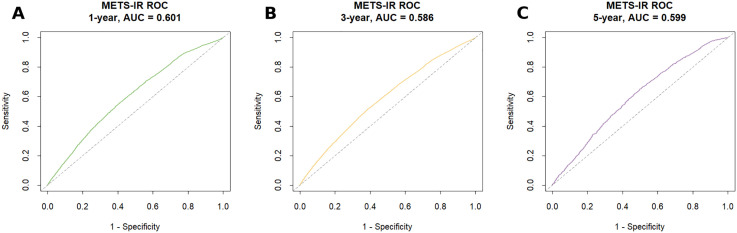
Time-dependent AUC at 1 (A), 3 (B), and 5 years (C). Adjusted for age, gender, marital status, SBP, DBP, LDL-C, BMI, WC, frequency of exercise, alcohol consumption status, and smoking status. Abbreviations: METS-IR, the metabolic score for insulin resistance; ROC, receiver operating characteristic; AUC, area under the curve.

Through time-dependent ROC curve analysis, we assessed the predictive ability of METS-IR for diabetes at different time points. The results showed that the AUC values were 0.601, 0.586, and 0.599 for 1 year, 3 years, and 5 years (see Fig 4).

## Discussion

This study systematically investigated the relationship between METS-IR and the risk of developing diabetes based on a large prospective cohort of rural Chinese adults. It is important to note that while our data did not clinically distinguish between type 1 and type 2 diabetes, the vast majority of incident diabetes in adult populations is type 2. Therefore, our research results are also applicable to studies based on T2DM risk prediction. The results showed that there was a significant positive association between METS-IR levels and the onset of diabetes and a nonlinear dose-response relationship. Specifically, the RCS curve displayed an approximately monotonic increase in diabetes risk with rising METS-IR levels, with a steeper slope observed in the lower to moderate METS-IR range, suggesting that initial increments in METS-IR are associated with a more pronounced rise in diabetes risk. At higher METS-IR levels, the curve plateaued somewhat, indicating a potentially attenuated risk increase beyond a certain threshold. This plateauing effect may reflect risk saturation at extreme insulin resistance levels, measurement ceiling effects of the METS-IR index, or selective survival bias in this high-risk population. Notably, our findings further corroborate and extend the accumulating evidence that METS-IR is a practical indicator of diabetes risk. Kaplan-Meier curves showed that the cumulative incidence of diabetes increased progressively with increasing levels of METS-IR. Given that no statistically significant interactions were detected, subgroup analyses were conducted primarily to assess the consistency of the association rather than to identify effect modification. Stratified analyses showed that the modified association remained consistent across all subgroups. The strength of association varied across subgroups, but these exploratory findings should be interpreted with caution, as they may be influenced by residual confounding or the play of chance. The results of the sensitivity analysis further supported the robustness of the main analysis. The direction and strength of the association between METS-IR and the risk of developing diabetes remained consistent after excluding individuals with BMI < 18.5, follow-up time <24 months, and severe chronic diseases at baseline.

METS-IR is a novel metabolic index for assessing insulin resistance (IR) without the need for direct insulin measurement [16]. It integrates fasting plasma glucose (FPG), lipid parameters, and obesity-related indicators and possesses the advantages of simple operation, low cost, and easy dissemination, which makes it particularly suitable for resource-limited primary care settings and large-scale epidemiological investigations [26]. Compared with the traditional insulin-based assessment of insulin resistance (IR), METS-IR is more practical and scalable. Non-insulin IR indices such as TyG, TyG-BMI, and TG/HDL-C proposed in earlier studies have been widely used for IR assessment and have been shown to be positively associated with diabetes risk [27–31]. The findings of the present study are consistent with previous studies that also found a positive association between METS-IR and new-onset diabetes [16,32–34]. It has been shown that the risk of diabetes rises by approximately 80% for each SD increase in METS-IR in a rural Chinese population [35]. In addition, a prospective cohort study of Chinese middle-aged and elderly people without T2DM at baseline in METS-IR names showed that an increase in SD units of baseline METS-IR was significantly associated with the first occurrence of T2DM, with the risk of new-onset T2DM in the highest quartile of the METS-IR group being 2.72 times higher than that in the lowest quartile among all participants [26]. Another study based on multicenter physical examination data also found that, after adjusting for possible confounding factors, METS-IR was significantly associated with new-onset diabetes (HR = 1.077; 95%CI: 1.073–1.082, P < 0.0001), among which the risk of diabetes onset in the fourth quartile group was 6.261-fold that of the first quartile group [36]. In the China Health and Retirement Longitudinal Study, 4,031 people ≥45 years of age were followed up, and the cumulative METS-IR was used to reflect long-term exposure levels. The results showed that the risk of diabetes development was significantly higher for each standard deviation increase in cumulative METS-IR, and the association remained robust in multivariate models [37]. The study also revealed a dose-response relationship between METS-IR and diabetes; that is, as the level of METS-IR increases, the risk of diabetes gradually rises. This trend has been verified in different populations. It included 12,107 rural Chinese residents [35], 12,290 non-obese Japanese adults [34], and 14,699 American adults [33].

In addition, regarding the predictive ability, the results of a retrospective cohort study showed that the AUC of METS-IR for the prediction of diabetes at 3, 4, and 5 years was 0.729, 0.718, and 0.720, respectively [36]. However, in the present study, the AUC values of METS-IR at 1, 3, and 5 years were 0.601, 0.586, and 0.599. In our research, the predictive power is more conservative than the results of the above-mentioned studies. This kind of change is very common and is often influenced by the characteristics of the population, queue design, and model adjustment strategies. However, the consistent but modest predictive ability demonstrated here supports its potential role in early risk warning, especially considering its practical advantages of simplicity and low cost. In the future, METS-IR can be used as a simple and useful indicator to assess the risk of diabetes at the population level, but it is not sufficient for individual-level risk prediction or as an independent screening tool in clinical practice by itself.

The potential mechanisms underlying the association between METS-IR and the risk of developing diabetes are not yet fully defined, and several possible mechanisms have been proposed to explain this association. First, insulin resistance (IR) usually precedes the clinical onset of diabetes and plays a key pathological role early in the disease [15]. Hyperglycemia can increase reactive oxygen species (ROS) levels in pancreatic β-cells through multiple metabolic pathways, and β-cells themselves are weak in antioxidant capacity and susceptible to oxidative stress damage. Continuous accumulation of ROS leads to β-cell dysfunction and even apoptosis, inhibiting insulin secretion and thus accelerating the onset and development of diabetes [34,38,39]. Secondly, abnormal accumulation of adipose tissue, especially visceral fat accumulation in the liver and pancreas, is considered to be an important factor leading to insulin resistance. This fat deposition may interfere with insulin action and signaling pathways, disrupting the homeostasis of glucose and lipid metabolism and leading to further elevation of blood glucose and lipid levels [40]. In addition, high triglyceride levels may increase free fatty acids (FFA), impair insulin signaling pathways in skeletal muscle and liver, and induce oxidative stress [41]; whereas decreased HDL-C levels weaken anti-inflammatory and antioxidant capacities, thereby promoting insulin resistance [42]. In conclusion, METS-IR, as a comprehensive indicator of glycemic, lipid, and obesity status, may mediate insulin resistance through mechanisms of oxidative stress, lipotoxicity, and metabolic disorders, thereby increasing the risk of diabetes. This hypothesis still needs to be tested by more basic and clinical studies.

Notably, this study retrospectively investigated the association between METS-IR levels and the risk of diabetes mellitus based on a large dynamic cohort from a rural area in central China. The study used a restricted cubic spline model to fit the dose-response relationship between METS-IR levels and the risk of diabetes onset, making the association between the two more intuitive and accurate. We also carried out a multisubgroup analysis to assess the differences in the effects of METS-IR among people with different ages, genders, marital statuses, exercise frequencies, and drinking statuses. The results showed consistent positive associations in all subgroups, further supporting its potential as a risk stratification indicator. In addition, the study included METS-IR as a categorical and continuous variable in the model, respectively, and carried out sensitivity analyses to validate the robustness of the main results. Meanwhile, the predictive ability of METS-IR for the onset of diabetes at 1 year, 3 years, and 5 years was evaluated through time-dependent ROC curve analysis. Based on this, the AUC value and 95%CI were calculated, indicating that its predictive ability in the early risk identification of diabetes is relatively conservative, and its discriminatory ability as an independent screening or diagnostic tool is limited. This reinforces the conclusion that, while potentially useful for population-level risk stratification, METS-IR alone offers insufficient accuracy to guide clinical decisions for individuals. Strict quality control was performed throughout the design and implementation stages of this study to ensure the authenticity of the data and the reliability of the conclusions. Despite the multifaceted strengths of this study, certain limitations still exist. First, a very large proportion of the initial population was excluded, mainly due to having only one examination record, which may introduce selection bias. Second, the definition of diabetes relied on FPG, HbA1c, or self-report, without routine Oral Glucose Tolerance Tests (OGTT), potentially leading to an underestimation of incidence, particularly of isolated postprandial hyperglycemia. Moreover, as data were derived from annual health checkups, these annual assessments may have missed short-term glycemic fluctuations, potentially biasing the results toward the null. It should also be noted that our data do not distinguish between types of diabetes. Third, while we adjusted for a wide range of confounders, residual confounding from unmeasured factors such as dietary patterns, family history of diabetes, or medication use (e.g., statins, thiazides, or beta-blockers) cannot be entirely ruled out; the absence of these data may have led to an overestimation of the observed association. Fourth, regarding our covariate adjustment strategy, the inclusion of both BMI and waist circumference in the fully adjusted model (Model III), while necessary to address potential confounding by these established risk factors, may also introduce partial overadjustment or collinearity given their conceptual overlap with components of METS-IR. Consequently, the hazard ratio estimated in Model III should be interpreted as a potentially conservative estimate of the independent association between METS-IR and diabetes risk. Finally, as the study population was only from a rural population in central China, and the cohort was drawn from an annual health check-up program, the representativeness of the sample was limited, and the extrapolation of the results still needs to be further verified in other populations.

## Conclusions

In this study, we found that elevated METS-IR levels significantly increased the risk of diabetes in rural adults in China, and there was a nonlinear positive dose-response relationship between the two. This indicates that METS-IR is an important risk predictor for the onset of diabetes. Although its discriminatory ability as an independent screening tool is limited, considering its practical advantages of simplicity, non-invasiveness, and low cost, METS-IR is suitable for the initial risk stratification of primary health care institutions with limited resources. Future research should focus on combining METS-IR with other readily available risk factors to develop more powerful predictive models and explore their utility in different populations and intervention strategies.

## Supporting information

S1 Fig Screening flowchart of participants. (PDF)

S1 Table Distribution of alternative insulin resistance indices across METS-IR quartiles at baseline. (DOCX)

S2 Table Sensitivity analysis of METS-IR levels and the risk of diabetes onset. (DOCX)
