# Supplementary figures and images for: The relationship between METS-IR and the risk of diabetes incidence in rural adults in China: A retrospective cohort study based on dynamic population
Source: PLoS One. 2026 Jan 28;21(1):e0341612. doi: 10.1371/journal.pone.0341612 (PMC12851496; doi:10.1371/journal.pone.0341612)

S1 Figure Screening flowchart of participants

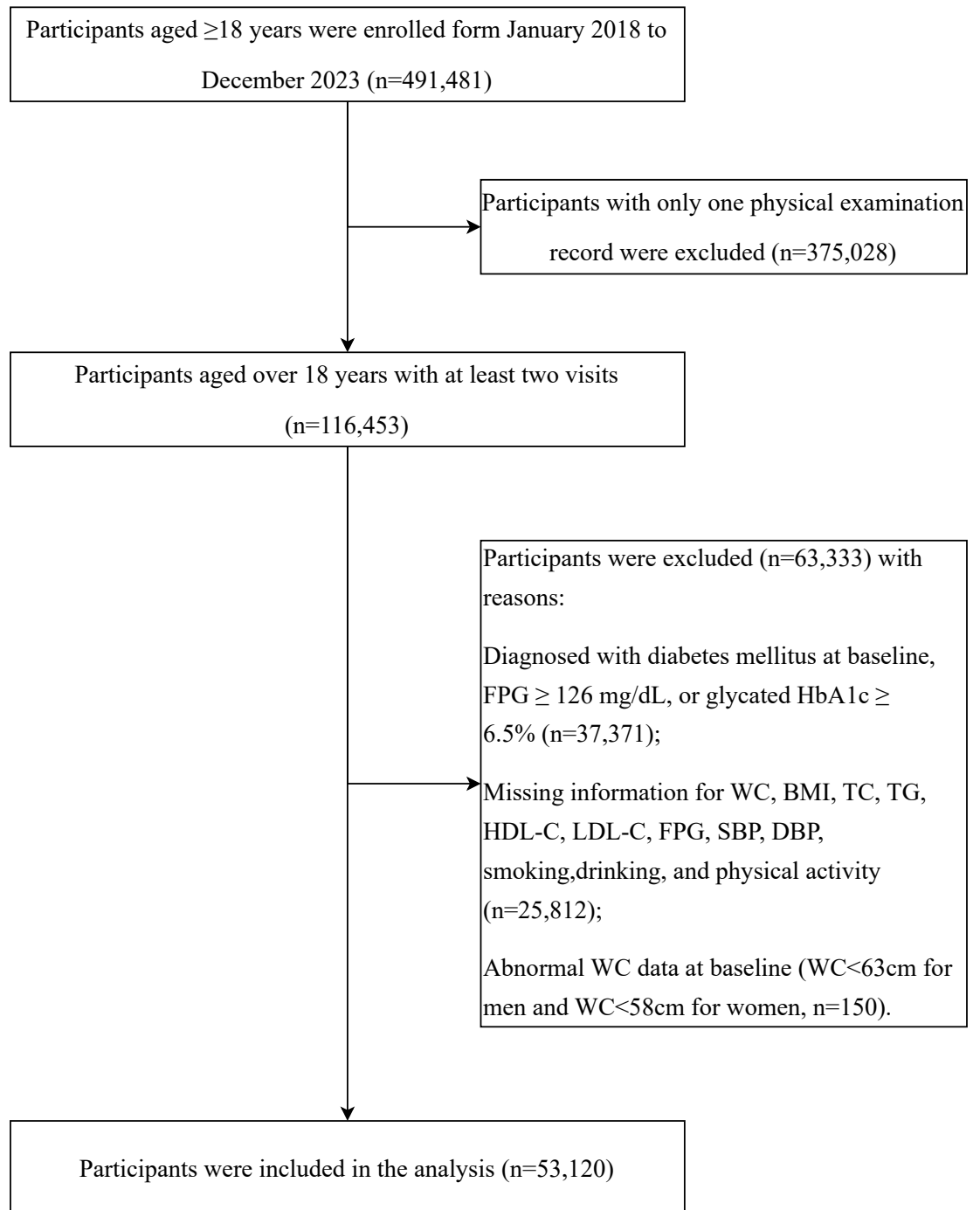

Supplement: S1 Fig — (PDF) [file pone.0341612.s001.pdf]
