# Supplementary material for: The relationship between METS-IR and the risk of diabetes incidence in rural adults in China: A retrospective cohort study based on dynamic population
Source: PLoS One. 2026 Jan 28;21(1):e0341612. doi: 10.1371/journal.pone.0341612 (PMC12851496; doi:10.1371/journal.pone.0341612)
Supplement: S1 Table — (DOCX) [file pone.0341612.s002.docx]

**S1 Table.** Distribution of alternative insulin resistance indices across METS-IR quartiles at baseline

|  | METS-IR quartiles | | | |  |
| --- | --- | --- | --- | --- | --- |
| Variables | Q1(n=13280) | Q2(n=13280) | Q3(n=13280) | Q4(n=13280) | *P* value |
| TyG | 8.2 (7.9, 8.5) | 8.4 (8.1, 8.7) | 8.6 (8.3, 8.9) | 8.8 (8.5, 9.2) | <0.001 |
| TyG-BMI | 172.8 (161.5, 183.7) | 198.4 (189.5, 207.9) | 219.8 (210.0, 230.4) | 251.0 (237.1, 268.8) | <0.001 |
| TyG-WC | 648.2 (605.6, 691.6) | 704.9 (665.5, 746.1) | 753.6 (712.9, 797.9) | 829.4 (776.9, 888.0) | <0.001 |
| LAP | 16.5 (10.7, 24.4) | 25.3 (17.8, 35.8) | 35.4 (25.4, 49.3) | 56.0 (39.3, 80.0) | <0.001 |
| VAI | 1.0 (0.7, 1.4) | 1.3 (0.9, 1.9) | 1.7 (1.2, 2.4) | 2.5 (1.7, 3.9) | <0.001 |
| TG/HDL-C | 0.6 (0.4, 0.8) | 0.8 (0.6, 1.1) | 1.1 (0.8, 1.5) | 1.6 (1.1, 2.3) | <0.001 |

Abbreviations: TyG, triglyceride glucose index; TyG-BMI, TyG-body mass index; TyG-WC, TyG-waist circumference; LAP, lipid accumulation product; VAI, visceral adiposity index; TG/HDL-C, triglyceride-to-high-density lipoprotein cholesterol ratio.
