# Supplementary material for: The relationship between METS-IR and the risk of diabetes incidence in rural adults in China: A retrospective cohort study based on dynamic population
Source: PLoS One. 2026 Jan 28;21(1):e0341612. doi: 10.1371/journal.pone.0341612 (PMC12851496; doi:10.1371/journal.pone.0341612)
Supplement: S2 Table — (DOCX) [file pone.0341612.s003.docx]

**S2 Table.** Sensitivity analysis of METS-IR levels and the risk of diabetes onset.

|  | METS-IR  (per SD increase) | Q1 | Q2 | Q3 | Q4 | *P* value |
| --- | --- | --- | --- | --- | --- | --- |
| Sensitivity-1 |  |  |  |  |  |  |
| Crude model | 1.11 (1.1, 1.13) | Ref | 1.19 (1.13, 1.25) | 1.32 (1.26, 1.39) | 1.51 (1.43, 1.58) | <0.001 |
| model Ⅰ | 1.13 (1.12, 1.14) | Ref | 1.22 (1.16, 1.28) | 1.39 (1.32, 1.46) | 1.63 (1.55, 1.71) | <0.001 |
| model Ⅱ | 1.12 (1.11, 1.14) | Ref | 1.21 (1.15, 1.27) | 1.36 (1.3, 1.43) | 1.59 (1.51, 1.67) | <0.001 |
| model Ⅲ | 1.09 (1.07, 1.11) | Ref | 1.2 (1.14, 1.26) | 1.3 (1.22, 1.38) | 1.44 (1.34, 1.54) | <0.001 |
| Sensitivity-2 |  |  |  |  |  |  |
| Crude model | 1.11 (1.09, 1.13) | Ref | 1.23 (1.14, 1.32) | 1.33 (1.24, 1.42) | 1.51 (1.41, 1.62) | <0.001 |
| model Ⅰ | 1.12 (1.11, 1.14) | Ref | 1.25 (1.17, 1.35) | 1.38 (1.29, 1.48) | 1.62 (1.51, 1.74) | <0.001 |
| model Ⅱ | 1.12 (1.1, 1.14) | Ref | 1.25 (1.16, 1.34) | 1.37 (1.27, 1.47) | 1.59 (1.48, 1.71) | <0.001 |
| model Ⅲ | 1.09 (1.07, 1.11) | Ref | 1.22 (1.13, 1.31) | 1.29 (1.18, 1.4) | 1.43 (1.3, 1.58) | <0.001 |
| Sensitivity-3 |  |  |  |  |  |  |
| Crude model | 1.19 (1.12, 1.26) | Ref | 1.16 (0.98, 1.38) | 1.28 (1.07, 1.52) | 1.62 (1.34, 1.95) | <0.001 |
| model Ⅰ | 1.18 (1.11, 1.25) | Ref | 1.15 (0.97, 1.36) | 1.26 (1.05, 1.5) | 1.59 (1.32, 1.92) | <0.001 |
| model Ⅱ | 1.16 (1.1, 1.24) | Ref | 1.12 (0.94, 1.33) | 1.21 (1.01, 1.45) | 1.51 (1.25, 1.83) | <0.001 |
| model Ⅲ | 1.13 (1.04, 1.24) | Ref | 1.09 (0.91, 1.31) | 1.17 (0.94, 1.46) | 1.35 (1.03, 1.78) | 0.032 |

Sensitivity analysis-1: subjects with a BMI <18.5 kg/m² were excluded.

Sensitivity analysis-2: on the basis of sensitivity analysis-1, subjects with a follow-up period of less than 24 months were further excluded.

Sensitivity analysis-3: on the basis of sensitivity analysis-2, further excluded subjects with hypertension, chronic obstructive pulmonary disease, coronary heart disease, stroke, cancer, and severe mental illness at baseline.

Crude Model: unadjusted for any variables.

Model I: adjusted for age, gender, and marital status.

Model II: adjusted for age, gender, marital status, SBP, DBP, and LDL-C.

Model III: adjusted for age, gender, marital status, SBP, DBP, LDL-C, BMI, WC, frequency of exercise, alcohol consumption status, and smoking status.

Abbreviations: BMI, body mass index; WC, waist circumference; SBP, systolic blood pressure; DBP, diastolic blood pressure; LDL-C, low-density lipoprotein cholesterol; METS-IR, the metabolic score for insulin resistance.
